# Enhancing Hydraulic Lime Mortar with Metakaolin: A Study on Improving Restoration Materials for Historic Buildings

**DOI:** 10.3390/ma17143548

**Published:** 2024-07-18

**Authors:** Xiaolong Wang, Huaishuai Shang, Junhao Zhou, Lilong Gu, Zhenhao Xiao, Xiaoqin Wang, Ruiping Wang

**Affiliations:** 1 School of Civil Engineering, Qingdao University of Technology, Qingdao 266033, China; wangxiaolong200101@163.com (X.W.);; 2 Zhongqing Jianan Group, Qingdao 266011, China

**Keywords:** restoration materials, hydraulic lime mortar, metakaolin, mechanistic analysis, microstructures, water vapor transmission

## Abstract

This study investigates the enhancement of hydraulic lime mortar (HLM) using varying contents of metakaolin (MK) to improve its application in the restoration of historic buildings. Samples from historic structures were analyzed, and the effects of different MK contents on the physical and mechanical properties of HLM were examined. The reaction mechanism and microstructural changes were evaluated using XRD and SEM analysis. The results indicated that increasing MK levels in HLM led to a decrease in fluidity, with fluidity reducing by 4.8% at 12% MK. The addition of MK increased water consumption for standard consistency by 5.4% and shortened the final setting time by 10.2%. MK consumption promoted secondary hydration, enhancing compressive strength by up to 98.1% and flexural strength by up to 55.1%, and increasing bonding strength by 26.9%. The density of HLM improved with MK addition, slightly reducing moisture content by 4.5% and water absorption by 4.6%, while the water vapor transmission properties decreased by 50.9%, indicating reduced porosity. The elastic modulus of the mortar increased significantly from 2.19 GPa to 7.88 GPa with the addition of MK, enhancing rigidity and crack resistance. The optimal blend for restoration materials was found to be 9.0% MK and 25.0% heavy calcium carbonate and was characterized by moderate mechanical strength, enhanced early strength, commendable permeability, minimal risk of cracking, and ease of application. This blend is highly suitable for the rehabilitation of historic structures.

## 1. Introduction

Urban construction faces specific challenges amidst accelerating urbanization, particularly in preserving non-renewable and valuable historic structures that epitomize a city’s heritage and cultural identity [1]. Protecting these edifices is essential in safeguarding the city’s historic memory [2] and ensuring that its unique cultural ambiance persists [3]. The restoration of historic architecture has been explicitly suggested and underlined in national policy papers as a crucial component of urban development projects [4].

Lime mortar is highly effective in the restoration of historic architecture due to its compatibility with older materials and its ability to allow structures to breathe [5]. This breathing property helps prevent moisture retention, which could otherwise cause structural damage. Lime mortar has been widely used for centuries due to its role in preserving the authenticity of historic buildings [6]. Its flexibility accommodates slight movements in structures without cracking, which is essential for long-term preservation [7].

Despite their historical significance, lime mortars exhibit certain weaknesses, such as pronounced porosity and limited early strength, potentially impacting restoration efficacy. These drawbacks can affect the restorative outcome. Hydraulic lime material has been increasingly used in place of pure lime to address these issues [8]. Hydraulic lime offers several advantages, such as quick setting, hardening [9], moderate strength [10], and good adhesion [11]. It has become a popular binder in North American restoration projects for its rapid setting compared to non-hydraulic hydrated lime mortars and its high water vapor transmissivity. Nonetheless, the exploration into hydraulic lime materials remains ongoing and lacks exhaustive understanding. Predicting the long-term performance of HLM is complex due to the diverse mineral compositions of these substances [12]. More research is needed to support the development of this material.

In practical engineering applications, the development of early strength in HLM is still slow [13], resulting in restoration effects that are not yet ideal; thus, its application is limited [14]. The performance of HLM needs to be improved by adding pozzolanic materials as an active ingredient to overcome these issues. These substances include, for instance, silica fume, MK, and rice husk ash [15]. These materials enhance the mechanical qualities and durability of the mortar [16].

Pozzolanic materials can efficiently inhibit the alkaline chemicals produced during material hydration. MK, a dehydrated alumino-silicate, typically appears white [17]. It has been investigated as an additive used in concrete materials and has improved the mechanical qualities and durability of materials well [18]. As an excellent pozzolanic material, its ability to react with Ca(OH)_2_, forming C-S(A)-H, improves the material qualities; thus, its use in concrete materials is recommended [19]. Similarly, HLM can also be used to utilize the high activity of MKs to enhance their performance. For historic buildings, MK’s high whiteness value aligns well with aesthetic needs, making it ideal for restoration work. Furthermore, a lot of MK is frequently gathered and kept in storage for extended periods of time, wasting valuable land resources. Many important mineral components are wasted, potentially affecting air quality and adding to air pollution [20]. Resource usage that is logical and effective is made possible by using MK as a raw material [10]. These benefits show that MK is a good choice for historic structure repair.

The utilization of MK in HLM has not been extensively studied in the past [21], and the exact mechanism of modification remains to be determined [22]. Most of the research conducted on MK has focused on cement mortar [23] or concrete materials [24]. This study specifically aimed to explore the effects of varying MK content on the physical phase composition and microstructure of HLM, as well as its influence on the physical and mechanical properties of HLM. The objective was to gain a deeper understanding of the mechanism of action and to offer guidance for the restoration of historic buildings.

## 2. Sampling and Analysis of Raw Materials for Historic Buildings

**Figure 1:**
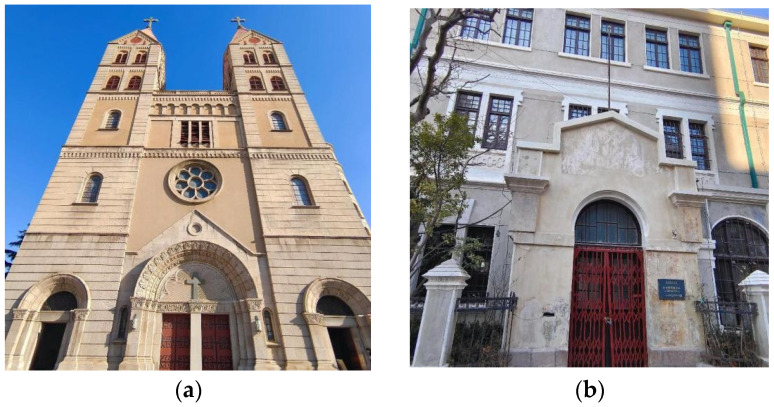
Historical buildings in the Qingdao: (**a**) the Qingdao Catholic Church; (**b**) the former site of Sacred Heart Convent.

**Figure 2:**
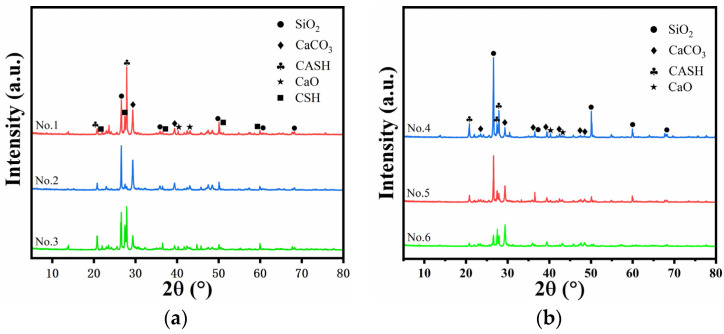
(**a**) XRD patterns of the Qingdao Catholic Church (No.1/No.2/No.3); (**b**) XRD patterns of the former site of Sacred Heart Convent (No.4/No.5/No.6).

In Qingdao, the brick and stone binding materials of several historic buildings, including the Qingdao Catholic Church (see Figure 1a) and the former site of the Sacred Heart Convent (see Figure 1b), were analyzed using X-ray diffraction (XRD), and the results are displayed in Figure 2. The Qingdao Catholic Church, a unique structure designed by the German architect Bialucha in a blend of Gothic and Roman architectural styles, was completed in 1932. The Sacred Heart Convent, built in 1902, features the typical architectural style of southern Germany.

The raw materials of the ancient structures in Qingdao were subjected to XRD examination, which indicated that the diffraction peak of SiO_2_, which mostly correlates with the fine aggregates in the construction materials, has a diffraction angle of roughly 26°. The diffraction angle at about 27° to 28° is dominated by the diffraction peak of the C-(A)-S-H gel, corresponding to the hydration products in the material. The diffraction peak of CaCO_3_, whose origin is primarily the carbonization of hydration products in building materials and the carbonization of air-hardened components, dominates the diffraction angle of about 28°to 29°. The graph illustrates how the composition and content of various construction materials can differ substantially. The materials all include calcium carbonate and silicate and are therefore consistent with the physical properties of HLM. It was discovered that white cement and hydrated lime were employed as the primary raw materials to design the restoration materials when combined with the material aesthetic features.

## 3. Materials and the Mixing Proportion

### 3.1. Raw Materials

#### 3.1.1. White Cement

**Figure 3:**
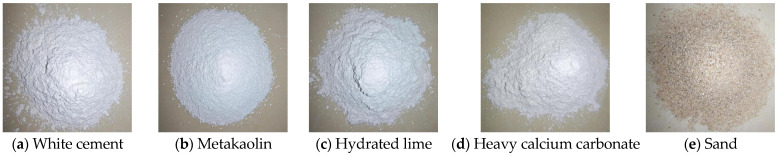
Raw materials morphology for testing.

The cement selected for this study is the Alba brand white Portland cement (see Figure 3a). The type is P.W 32.5. The cement index and the primary chemical composition of the material are detailed in Table 1 and Table 2.

#### 3.1.2. Metakaolin

The chosen MK (see Figure 3b) has a mesh size of 1250, a whiteness of 91%, and a 28-day pozzolanic activity index of 115 and is a highly reactive MK. According to the oxide composition test findings, the combined content of SiO_2_ and Al_2_O_3_ is 97.10%, with minimal impurity element concentration. The specific material data are presented in Table 3 below.

#### 3.1.3. Hydrated Lime 

Hydrated lime stands as one of the most exceptional options for restoration materials. It is an air-hardened substance with high whiteness, and its strength is crucial for the restoration of historic structures as it meets the reversibility standards of construction materials. The primary composition of the material is detailed in Table 4. In this study, the lime material (see Figure 3c) used contains 93.46% Ca(OH)_2_, and its 200-mesh passing rate is 96.14%.

#### 3.1.4. Heavy Calcium Carbonate

The heavy calcium carbonate (see Figure 3d) used in this study contains 98.5% CaCO_3_, with a mesh size of 325 and a whiteness of 95.51%.

#### 3.1.5. Sand

The selected fine river sand (see Figure 3e) had particle sizes no larger than 0.5 mm and an apparent density of 2543.10 kg/m^3^. Given that the test sand was fine, the muck content could significantly impact the test results. Therefore, the test sands were pre-washed and dried prior to use.

### 3.2. Design of Mixing Proportion

During pre-tests, it was observed that an excessive amount of lime admixture could significantly impact the workability of cementitious materials when the water–cement ratio was 0.6. On the other hand, an excessively high cement content can affect the desired restoration effect of the mortar. Silva et al. [25] concluded that an HLM with a cement admixture ranging from 25.0% to 50.0% exhibits good compatibility with historic buildings and provides satisfactory early strength when used as a restoration material. Considering all the factors, the cement content for this experiment was set at 35.0%. When the cement admixture was 35.0%, the fluidity of the HLM was only 141 mm. Heavy calcium carbonate was used in place of some of the lime to significantly improve the material’s workability, increasing the fluidity to 196 mm at a 25.0% heavy calcium carbonate content. However, the preliminary test findings suggested that the MK addition may make the HLM less fluid. Hence, some advanced fluidity allowance was provided. 

In this study, the control group is designated as LPSMK0 and consists of 35.0% white cement, 40.0% lime, and 25.0% heavy calcium carbonate, with no addition of metakaolin (MK). The cement–sand ratio is maintained at 1:2, and the water–cement ratio is 0.60. This control group serves as a baseline to compare the effects of the varying MK content in the experimental groups. The experimental groups (LPSMK1, LPSMK2, LPSMK3, and LPSMK4) include 3.0%, 6.0%, 9.0%, and 12.0% MK, respectively, while the lime content is adjusted accordingly. Table 5 displays the mixing proportions for this experiment.

### 3.3. Sample Preparation and Curing Process

The preparation of the mortar samples followed a standardized procedure to ensure consistency across all the tests. Initially, the dry components (white cement, hydrated lime, heavy calcium carbonate, and metakaolin) were weighed and thoroughly mixed to achieve a uniform blend. The water required was then gradually added to the dry mixture while being continuously stirred to avoid lump formation. The mixing was performed using a mechanical mixer at a low speed for 30 s, followed by a high-speed mix for 90 s to ensure homogeneity.

The prepared mortar was then poured into molds for different tests. After casting, the molds were vibrated to remove any entrapped air and to ensure a dense packing of the mortar. The samples were demolded after 24 h and cured under natural conditions to more closely simulate actual engineering environments.

## 4. Effect of MK Content on the Physical Properties of HLM

### 4.1. Fluidity

**Figure 4:**
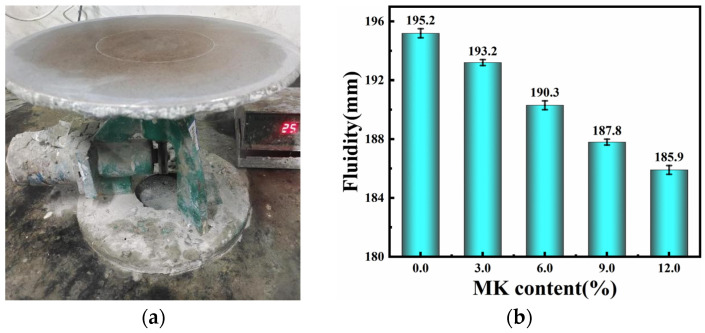
(**a**) NLD-3 type cement fluidity meter; (**b**) fluidity of HLM with different contents of MK.

The fluidity test was conducted according to the standard GB/T 2419-2005 [26], “Test Method for Fluidity of Cement Mortar”. The fluidity of HLM with varying MK content was assessed using an NLD-3 type cement fluidity meter (see Figure 4a); the results are presented in Figure 4b.

According to the experimental findings, the fluidity of the HLM decreased with increasing MK content at a water–cement ratio of 0.6. Compared to the control group (0% content), at 9% and 12% content, the fluidity of the mortar decreased by 3.8% and 4.8%, respectively. This trend is likely due to the high water-absorbent nature and fine granularity of MK [27]. The addition of MK alters the microstructure of the mortar, impacting its macroscopic physical properties [28]. This is because MK absorbs more free water in the substance since it has a more extensive specific surface area than lime. In addition, MK particles have irregular shapes and tend to agglomerate among themselves, resulting in a lack of a ball-bearing lubricating effect upon mixing, and this further increases the friction between particles [29].

### 4.2. Water Consumption for Standard Consistency and Setting Time

**Figure 5:**
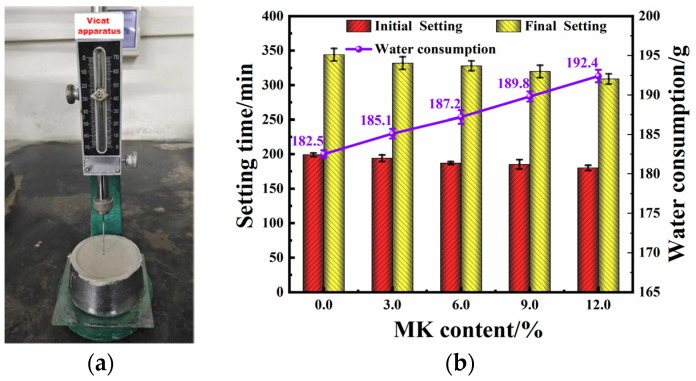
(**a**) Vicat apparatus; (**b**) water consumption of standard consistency and setting time of HLM with different contents of MK.

Based on the standard GB/T 1346-2011 [30], the water consumption for the standard consistency and the setting times of the different samples were measured using a Vicat apparatus [31] (see Figure 5a). The results of these measurements are displayed in Figure 5b.

The experiment demonstrated that adding MK increased the water consumption for the standard consistency of HLM and decreased the initial and final setting times. Compared to the control group (0% content), at 9% and 12% content, the initial setting time decreased by 7.0% and 9.5%, the final setting time decreased by 7.0% and 10.2%, and the water consumption increased by 4.0% and 5.4%, respectively. This experimental result is consistent with the fluidity test, and the increased water consumption results from MK’s higher specific surface area and increased particle friction and its amorphous structure [32]. After the addition of MK, the curing and setting time significantly reduced. This is mainly because the addition of MK promotes the hydration reaction of cement and can directly react with Ca(OH)_2_, quickly forming many hydration products with structural strength, thereby reducing the time required for curing and setting. Additionally, the filling effect of MK is also one of the reasons for the shortened setting time of HLM. Therefore, MK is beneficial for the solidification and hardening of cementitious materials [33].

### 4.3. Moisture Content and Water Absorption

**Figure 6:**
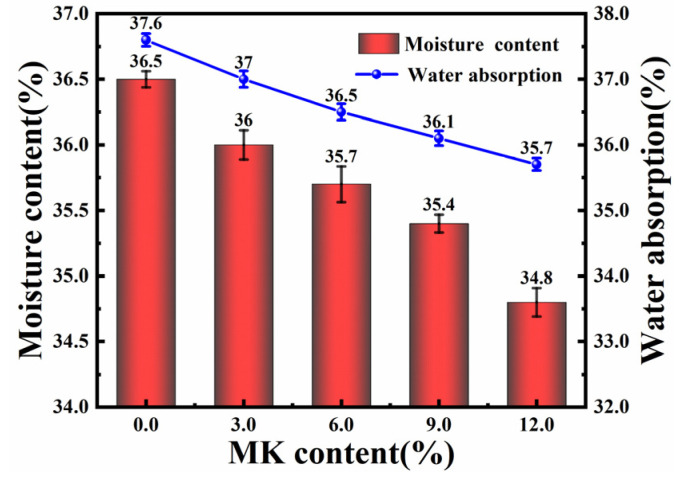
Moisture content and water absorption of HLM with different contents of MK.

In accordance with the “Standard for Test Methods of Basic Properties of Building Mortar” (JGJ/T 70-2009) [34], cubic specimens with dimensions of 70.7 mm × 70.7 mm × 70.7 mm were prepared for testing the moisture content and water absorption of the samples. At the age of 28 days, the initial mass of the sample was measured as *M*_0_. It was then dried in a constant-temperature drying oven until reaching a constant weight, and the mass was measured as *M_d_*. The sample was subsequently cooled to room temperature and soaked in water for 24 days. After wiping off the surface water, its mass was measured as *M_s_*. The sample’s moisture content and water absorption were calculated using Equations (1) and (2), respectively [35]. The test results are presented in Figure 6.

(1) $$M_{C}=\frac{\left(M_{0}-M_{d}\right)}{M_{0}}\times 100\%$$

(2) $$W_{a}=\frac{\left(M_{s}-M_{d}\right)}{M_{d}}\times 100\%$$

*M_c_*—moisture content of the sample.

*W_a_*—water absorption of the sample.

*M*_0_—the initial mass of the sample.

*M_d_*—the mass of the specimen after drying.

*M_s_*—the mass of the sample after full water absorption.

The moisture content and water absorption of the samples slightly decrease as the content of MK increases. Specifically, incorporating 6.0% MK results in a reduction of 2.1% in moisture content and a 2.4% decrease in water absorption. This trend intensifies with 12.0% MK, leading to a decrease of 4.5% in moisture content and 4.6% in water absorption. The likely reason behind this phenomenon is the substantial formation of hydration products due to MK addition, which renders the HLM denser and less porous [36]. Although the permeability and breathability levels of HLM modified with MK are reduced, they still maintain a relatively high level. Such characteristics are crucial for preserving the breathability and permeability of materials, especially in the restoration of historical structures where maintaining a moisture balance is key. The enhanced breathability of the material allows water movement through the structure, averting moisture accumulation within the walls and minimizing the risks of issues like condensation or dampness. Furthermore, this property helps prevent the accumulation of detrimental elements like moisture and mold within the walls [37]. The use of non-breathable, impermeable materials can interfere with the moisture balance in walls and accelerate their degradation. 

### 4.4. Mass Change

**Figure 7:**
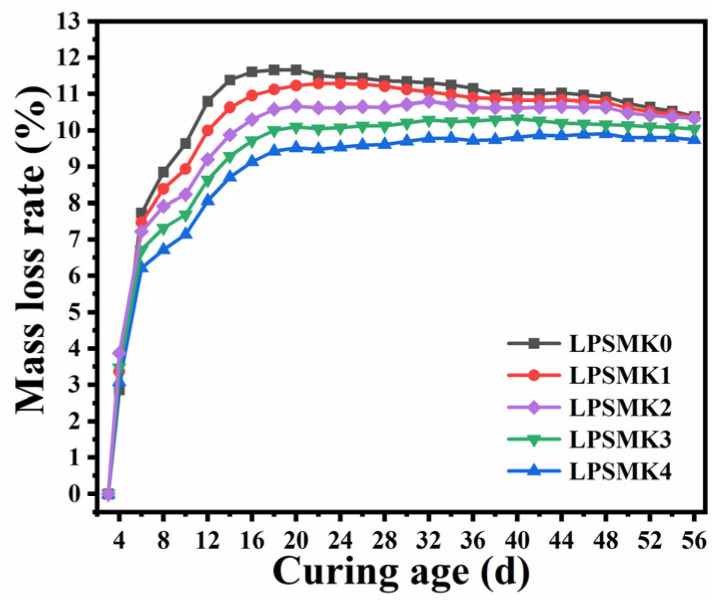
Mass loss rate of HLM with different contents of MK.

The mortar specimens were weighed immediately after demolding. Subsequently, the specimens were weighed every two days, and the rate of mass loss was calculated. The mass loss rate is defined as the percentage reduction in mass of a material over time [38]. It is a critical parameter in assessing the durability and longevity of materials in various conditions. In this context, the mass loss rate of the HLM specimens was tracked and plotted over the curing period, as illustrated in Figure 7. 

The mass change observed in the specimens LPSMK0 to LPSMK4 during a 56-day testing phase exhibits a unique trend of initial decrease followed by an increase. This pattern begins to shift at specific points in time for each specimen group: 18 days for LPSMK0, 24 days for LPSMK1, 32 days for LPSMK2, 40 days for LPSMK3, and 47 days for LPSMK4. The incorporation of MK in the mortar specimens significantly mitigates the initial mass loss. Additionally, MK’s presence delays the phase of mass regain in the mortar samples. Initially, there is a rapid mass loss post-demolding, which is primarily due to moisture evaporation and the resultant loss of unreacted water. As the curing progresses, a gradual mass recovery is observed in the specimens. This is attributed to two main factors: enhanced water absorption and retention capability due to the buildup of hydration products [39], and the carbonation reaction, which integrates atmospheric CO_2_ to form CaCO_3_, thereby increasing the specimen’s mass [40]. Notably, the rate of mass recovery is relatively slow, indicating a gradual carbonation process. With an increase in MK content, the rate of mass recovery consistently decreases. This slowdown is caused by the interaction of MK with lime and its pozzolanic reaction with Ca(OH)_2_ formed during cement hydration [41]. These reactions result in a reduced Ca(OH)_2_ concentration and a denser structural composition, collectively impeding the progression of the carbonation reactions. Thus, it can be inferred that higher MK content more significantly inhibits the carbonation process.

### 4.5. Drying Shrinkage

**Figure 8:**
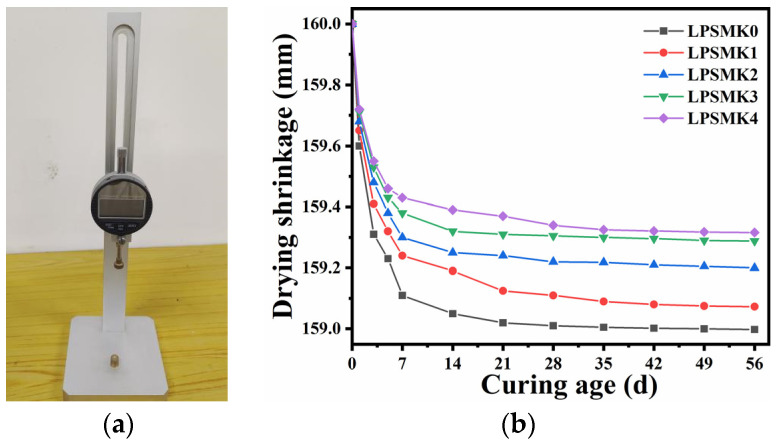
(**a**) A precision vertical mortar shrinkage device; (**b**) drying shrinkage of HLM with different contents of MK.

In accordance with the “Standard for Test Methods of Basic Properties of Construction Mortar” (JGJ/T 70-2009) [34], prismatic specimens with dimensions of 40 mm × 40 mm × 160 mm were prepared. Three sets of specimens were prepared for each group, and the arithmetic mean value was taken as the drying shrinkage value. The lengths of the specimens were gauged using a precision vertical mortar shrinkage device (see Figure 8a), calibrated to detect minute variations, to enhance measurement accuracy. The trend of dry shrinkage variation in the samples was determined by comparing the measured lengths with the starting lengths of the samples. Figure 8b presents the findings.

The experimental results indicate that the specimens’ shrinkage rate within 56 days follows a trend that is initially fast and then slow. The shrinkage rate of the specimens decreases with an increase in the dosage of MK. The total shrinkage rate of the specimens LPSMK3 to LPSMK5 is less than 0.5%, which significantly reduces the risk of drying shrinkage and cracking in the later stages. Upon closer scrutiny of the results, it becomes apparent that the integration of MK plays a pivotal role in the densification process of the HLM. The MK reacts to form a complex array of hydration products that occupy the void spaces within the mortar, effectively enhancing its compactness. This infilling mechanism is instrumental in diminishing the overall shrinkage rate, thereby augmenting the material’s resistance to the tensile stresses that lead to cracking [42]. The synergy between MK and the mortar’s constituents catalyzes a more robust and cohesive internal structure, ultimately contributing to the longevity and durability of the restoration material.

### 4.6. Water Vapor Transmission Properties

**Figure 9:**
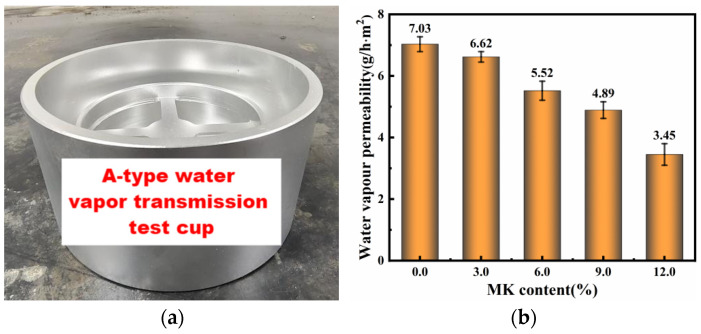
(**a**) A-type water vapor transmission test cup; (**b**) water vapor transmission properties of HLM with different contents of MK.

In this study, the water vapor transmission properties of hydraulic lime mortar (HLM) with different contents of metakaolin (MK) were evaluated to indirectly infer the porosity of the samples. The tests were conducted according to the “Test Methods for Water Vapour Transmission Properties of Building Materials and Products” (GB/T 17146-2015) [43]. Cylindrical disk samples with a diameter of 50 mm and a height of 20 mm were prepared for this purpose. The A-type water vapor transmission test cup was utilized for the measurements (see Figure 9a). Each sample was sealed in the test cup containing a desiccant and placed in a controlled environment chamber with a specified relative humidity. The weight of the test cup was measured periodically to determine the rate of water vapor transmission. The water vapor permeability for each sample was calculated based on the weight change over time. 

The results indicated variations in the transmission coefficients among the different MK content groups, as shown in Figure 9b. These variations provided an indirect measurement of the porosity of the HLM samples, correlating higher MK content with reduced transmission and suggesting a denser microstructure. Specifically, the water vapor transmission coefficient decreased from 7.03 g/(h·m^2^)) at 0% MK to 4.89 g/(h·m^2^)) at 9% MK and further to 3.45 g/(h·m^2^) at 12% MK, reflecting reductions of approximately 30.4% and 50.9%, respectively. This trend is consistent with the findings from the moisture content and water absorption tests presented in Figure 6, where both moisture content and water absorption decreased with increasing MK content. The densification of the mortar matrix due to the pozzolanic reaction of MK with Ca(OH)_2_, forming additional calcium silicate hydrates (C-S-H), fills the pore spaces and reduces the overall porosity of the mortar, thereby lowering the water vapor transmission.

## 5. Effect of MK Content on Mechanical Properties of HLM

### 5.1. Flexural and Compressive Strengths

**Figure 10:**
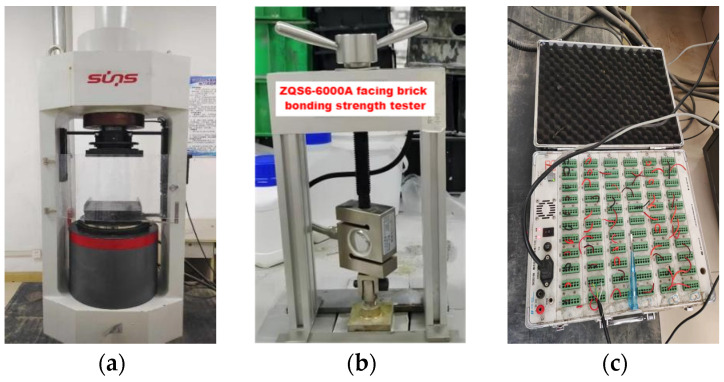
(**a**) YAW-3000 Microcomputer-Controlled Electro-Hydraulic Servo Testing Machine; (**b**) ZQS6-6000A facing brick bonding strength tester (Zhongke Beigong Group Ltd., Cangzhou, China); (**c**) DH3816 static stress and strain acquisition instrument (Donghua Group Ltd., Yancheng, China).

**Figure 11:**
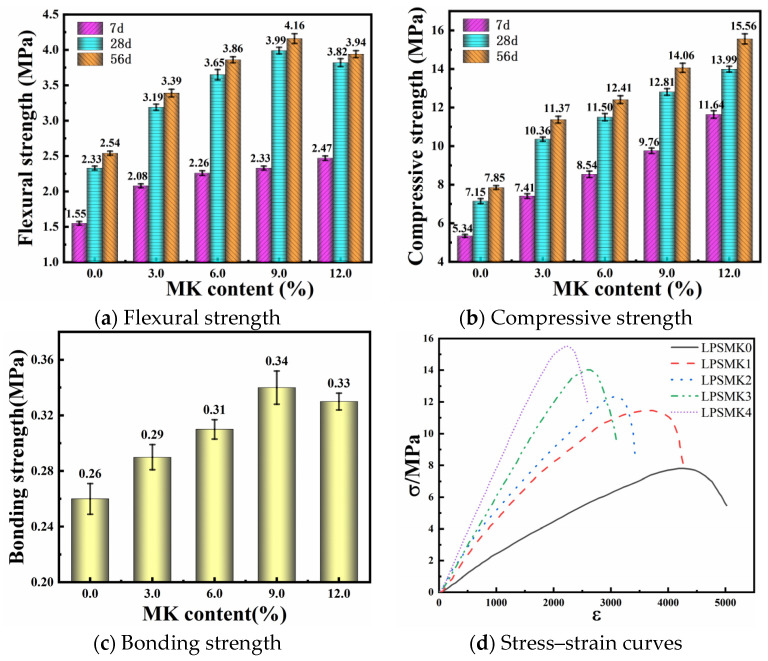
Mechanical properties of HLM with different contents of MK.

In accordance with the “Standard for Test Methods of Basic Properties of Construction Mortar” (JGJ/T 70-2009) [34], cubic specimens with dimensions of 70.7 mm × 70.7 mm × 70.7 mm were prepared. Three sets of specimens were prepared for each group, and the arithmetic mean of the three sets was taken as the cubic compressive strength of the group. In accordance with the “Test Method for Strength of Cement Mortar (ISO Method)” (GB/T 17671-2021) [44], prismatic specimens with dimensions of 40 mm × 40 mm × 160 mm were prepared. Three sets of specimens were prepared for each group, and the arithmetic mean of the three sets was taken as the flexural strength of the group. This study measures the compressive and flexural strength of mortar specimens using the YAW-3000 Microcomputer-Controlled Electro-Hydraulic Servo Testing Machine (Sansi Zongheng Group Ltd., Shenzhen, China) (see Figure 10a). The test results of the flexural and compressive strength for each group of mortar at 7 d, 28 d, and 56 d are shown in Figure 11a,b, respectively.

As shown in the figure, the results indicate that adding MK significantly enhances the flexural and compressive strength of the material under natural curing conditions. Simply incorporating a mere 3.0% of MK can effectively address the issue of insufficient early strength in HLM. After incorporating metakaolin into the material, the flexural and compressive strengths of the mortar significantly improved. Compared to the control group (0% content), at 9% and 12% content, the 56 d flexural strength of the mortar increased by 63.8% and 55.1%, respectively. Additionally, the 56 d compressive strength increased by 79.1% and 98.1%, respectively. This is mainly due to MK’s good pozzolanic properties, whose main mineral composition is SiO_2_ and Al_2_O_3_, which are poorly crystalline and cannot maintain a fixed form due to their lamellar structure [45]. These components consume Ca(OH)_2_ and actively participate in the hydration reaction, leading to increased secondary hydration of cement and the formation of stronger bonds within the material.

### 5.2. Bonding Strength

The bonding strength test method referred to the “Standard for Test Methods of Basic Properties of Construction Mortar” (JGJ/T 70-2009) [34]. Repair mortar bonding specimens with dimensions of 40 mm × 40 mm × 6 mm were prepared. Based on actual application conditions, the bonding strength test was conducted using a ZQS6-6000A facing brick bonding strength tester (see Figure 10b), with blue bricks as the substrate. The results of the bonding strength test of each group of mortar on the 28th day are shown in Figure 11c.

Incorporating metakaolin into the material initially improves the bonding strength of the mortar, but beyond a certain content, the strength begins to decrease. Compared to the control group (0% content), the bonding strength increased from 0.26 MPa to 0.34 MPa at 9% content, representing an increase of 30.8%. However, at 12% content, the bonding strength slightly decreased to 0.33 MPa, representing an increase of 26.9% from the control group but a slight decrease from the 9% content. This initial improvement can be attributed to the pozzolanic reaction of metakaolin, which enhances the formation of calcium silicate hydrate (C-S-H) gel, improving the microstructure and bonding properties of the mortar. The fine particles of metakaolin fill the pores within the mortar matrix, leading to a denser and more cohesive structure. However, when the content of metakaolin exceeds an optimal level, the excess fine particles may disrupt the balance of the mix, leading to reduced workability and potential issues with proper compaction and hydration, which in turn slightly reduce the bonding strength. This can result in insufficient contact between the mortar and masonry bricks, affecting the overall bonding strength.

### 5.3. Elastic Properties and Stress–Strain Curves

**Figure 12:**
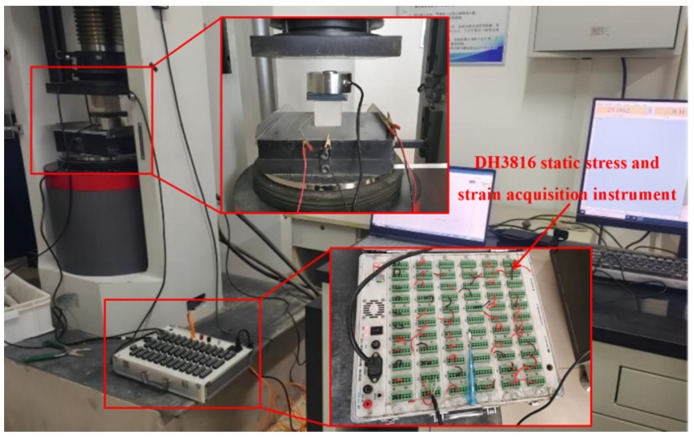
Elastic properties test.

The elastic properties and stress–strain curves of the specimens were evaluated following the guidelines of “Standard for Test Methods of Basic Properties of Construction Mortar” (JGJ/T 70-2009). Cube specimens, each with dimensions of 70.7 mm × 70.7 mm × 70.7 mm, were utilized. Strain gauges were affixed longitudinally and transversely at symmetrical positions along the midline on the sides of each specimen to record strain during compression. The testing was conducted using a YAW-3000 Microcomputer-Controlled Electro-Hydraulic Servo Testing Machine, with a load application rate of 0.8 kN/s. A pressure sensor was placed between the specimen and the testing machine to capture real-time axial pressure, ensuring accuracy. The DH3816 static stress and strain acquisition instrument (see Figure 10c) was used to gather data on stress and strain throughout the testing process. These tests provided valuable information on the mortar’s elastic modulus and Poisson’s ratio. The schematic diagram of the test is shown in Figure 12.

The elastic modulus refers to the constant ratio of longitudinal stress to longitudinal strain within the elastic deformation range of a material. Poisson’s ratio is defined as the ratio of lateral strain to longitudinal strain when the material is subjected to a uniformly distributed longitudinal force. The calculated results of the elastic modulus and Poisson’s ratio for the mortar materials are shown in Table 6. The elastic modulus for each group of samples is derived from Figure 11d; Poisson’s ratio is taken as the ratio of the lateral to longitudinal strain when the stress reaches 43% of the maximum stress during the normal operation of the mortar.

Figure 11d presents the stress–strain curves of the five groups of mortar samples. As the metakaolin content increases, the elastic modulus of the mortar significantly increases from 2.19 GPa to 7.88 GPa. Meanwhile, the Poisson’s ratio gradually decreases from 0.253 to 0.107, indicating improved rigidity and crack resistance. In the elastic phase, the addition of metakaolin significantly increases the slope of the stress–strain curve, and the stress and strain of the material show a linear relationship without visible cracks. Mortar samples with high metakaolin content exhibit higher rigidity in this phase. When the stress exceeds the elastic limit, the slope of the curve gradually decreases, and the material enters the plastic deformation phase, eventually reaching peak stress. Adding 9% and 12% metakaolin reduces the peak strain from 4178 to 2648 and 2207, respectively. The rapid decrease in stress after the peak in the samples with high metakaolin content indicates higher brittleness and lower ductility. This result is due to the increased density and reduced pore size from the formation of additional C-S-H gel, which enhances strength but also makes the material more brittle.

The addition of metakaolin (MK) significantly enhances the flexural, compressive, and bonding strengths of the mortar. Even the mortar with 3.0% MK content has achieved or surpassed the strength standard of European natural hydraulic lime NHL5. Compared to other ancient building materials mentioned in reference [46], such as burnt clay bricks, it also exhibits certain advantages. However, higher mechanical strength is not always desirable for historic building restoration, as excessive strength might lead to mechanical incompatibility with the ancient structure and fail to meet reversibility standards [25]. The sample with 9.0% MK content shows suitable strength for restoration purposes, providing adequate mechanical properties without compromising the structural integrity of historic buildings. Moreover, the addition of MK has been found to significantly improve bonding strength, ensuring better adhesion between mortar and masonry bricks.

## 6. Mechanistic Analysis 

### 6.1. XRD Analysis

**Figure 13:**
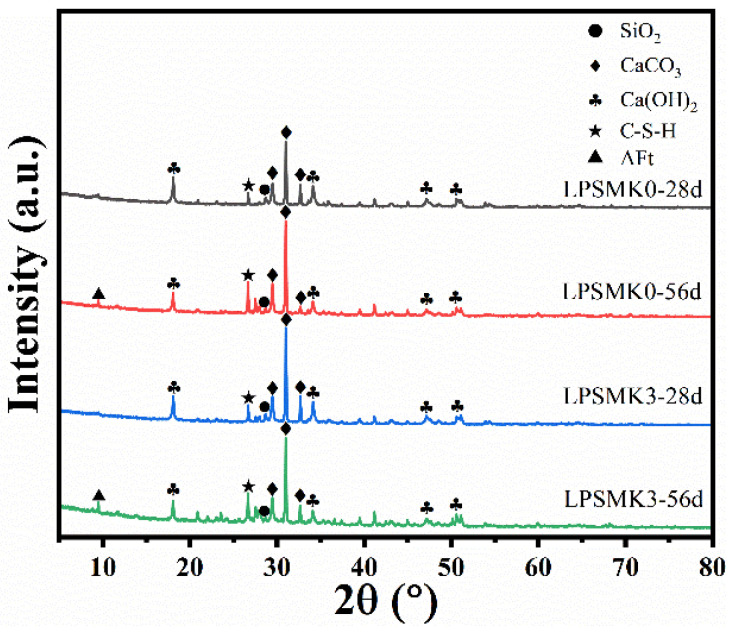
XRD patterns of samples LPSMK0 and LPSMK3 at 28 days and 56 days.

For XRD analysis, the mortar samples were cured for 28 and 56 days. Following the curing process, the samples were oven-dried at 60 °C to remove any residual moisture. The dried samples were then ground into a fine powder using a mechanical grinder. The ground samples were passed through a 200-mesh sieve to ensure uniform particle size. Approximately 1 g of the sieved powder was collected for analysis. The powdered samples were placed into an XRD sample holder, ensuring an even and compact surface. The XRD analysis was conducted to determine the phase composition of the mortar samples. The XRD patterns of the LPSMK0 and LPSMK3 mortars at 28 days and 56 days are shown in Figure 13.

According to the XRD examination findings, the sample’s major phases at 28 and 56 days of curing were composed of Ca(OH)_2_, CaCO_3_, SiO_2_, and C-S-H. The significant amount of SiO_2_ and Al_2_O_3_ in MK was mostly consumed. The strength of the C-S-H diffraction peak increased while the Ca(OH)_2_ diffraction peak weakened as the MK content increased. This is because the active SiO_2_ and Al_2_O_3_ directly react with lime, consuming Ca(OH)_2_ and facilitating cement hydration. The hydration process was mostly finished at 28 days, and the following rise in CaCO_3_ concentration is attributable to carbonation, according to a comparison of the XRD results at two ages. It was again demonstrated that the addition of MK could inhibit the carbonation of mortar. When compared with the original materials of the historic buildings, the prepared HLM is primarily composed of Ca(OH)_2_, C-S-H, CaCO_3_, SiO_2_, and AFt. Its main components are similar to those of the historic building materials. Moreover, the new product AFt does not affect the compatibility with the original structure, making it suitable for the restoration of historic buildings.

### 6.2. SEM Analysis

**Figure 14:**
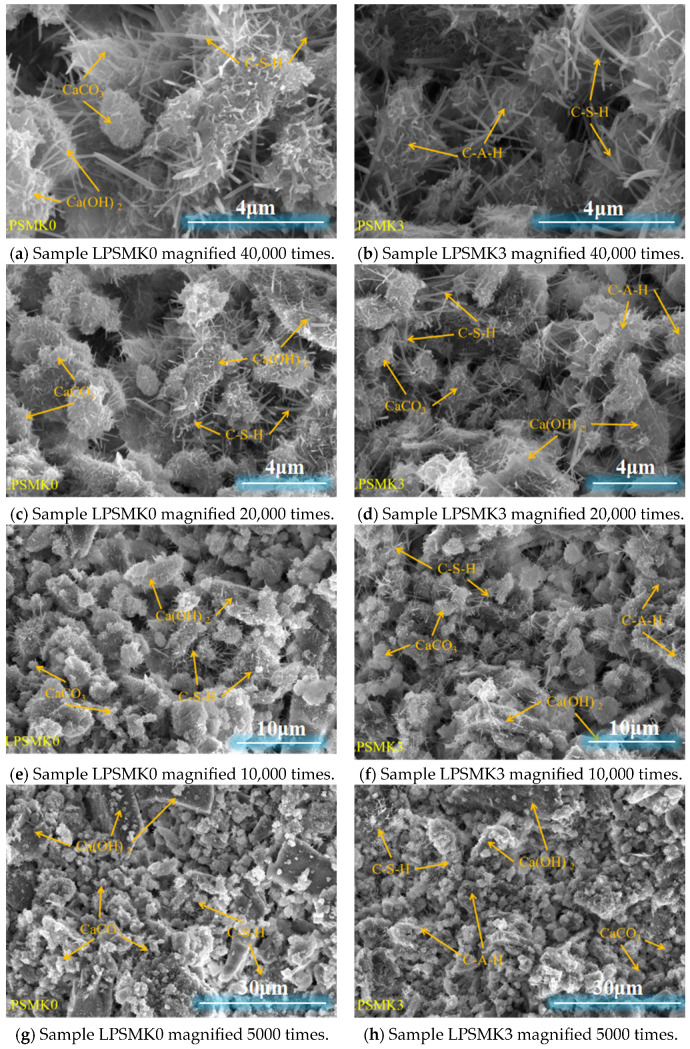
SEM images of samples LPSMK0 and LPSMK3 at 28 days.

The SEM analysis was conducted to observe the microstructural features of the mortar samples. Specimens measuring approximately 10 mm × 10 mm × 3 mm were prepared from the mortar cured for 28 days. These specimens were first oven-dried to remove any residual moisture. Following the drying process, the samples were fractured in liquid nitrogen to create a fresh surface for observation. The fractured samples were then coated with a thin layer of gold using a sputter coater to enhance their conductivity. The prepared samples were secured on aluminum stubs using double-sided carbon tape and subsequently analyzed using a scanning electron microscope [35]. This preparation method is straightforward and effective, ensuring the stability and integrity of the samples. Figure 14 illustrates the SEM photos of the LPSMK0 and LPSMK3 samples at 28 days.

As observed in Figure 14, compared to the LPSMK0 group without MK, the LPSMK3 group with 9.0% MK demonstrates notable changes in microstructure. Compared to the LPSMK0 specimen, the LPSMK3 specimen exhibits a reduction in the number of flake-like structures, a decrease in spherical structures, and a significant increase in needle-like structures. According to the literature [40], the flaky substances are identified as Ca(OH)_2_, the spherical substances as CaCO_3_, and the needle-like substances as C-S-H. These structures intertwine to form a spatial network, embedding small CaCO_3_ particles within the hydration products, thereby reducing pore size and densifying the microstructure. This finding suggests that increasing the MK content causes more complete hydration processes, which consume more Ca(OH)_2_ and produce more hydration products.

The LPSMK3 specimens without MK do not directly consume Ca(OH)_2_; hence, they do not inhibit the carbonation reaction. Flaky Ca(OH)_2_ carbonates to form spherical CaCO_3_, providing some mechanical strength during carbonation. However, incorporating MK consumes Ca(OH)_2_, restraining carbonation. The resultant C-S-H gel from this reaction has a higher mechanical strength than the strength provided by carbonated Ca(OH)_2_. The flocculent nature of the C-S-H gel significantly contributes to the enhancement of the strength. Therefore, the inclusion of MK in HLM significantly enhances its compressive and flexural strengths.

## 7. Conclusions

To determine the optimal ratio of restoration materials for historic buildings, samples from historic structures were analyzed in this study. Based on these analyses, the impact of different MK contents on the physical and mechanical properties of HLM was investigated. The reaction mechanism and microstructural changes were examined using XRD and SEM analysis, leading to the following conclusions.

(1) As the amount of MK increases in HLM, its fluidity decreases, and too much MK can adversely affect the mortar’s workability.
(2) The addition of MK increases the water consumption for standard consistency in HLM due to its large specific surface area, and it also shortens the setting time of the mortar.
(3) MK consumption in HLM promotes secondary hydration, enhancing compressive and flexural strength. The bonding strength of HLM increased with the addition of MK, reaching optimal performance at around 9% MK content.
(4) Adding MK can enhance the density of HLM, while slightly reducing its moisture content and water absorption. The water vapor transmission properties decreased, indicating reduced porosity.
(5) The elastic properties and stress–strain curves indicated that the elastic modulus of the mortar increased significantly with the addition of MK, enhancing the material’s rigidity and crack resistance.
(6) Incorporating 9.0% MK and 25.0% heavy calcium carbonate into HLM forms the optimal blend for restoration materials. This specific combination is characterized by its moderate mechanical strength, enhanced early strength, commendable permeability, minimal risk of cracking, and ease of application, making it well suited for the rehabilitation of historic structures.

This study has several limitations. The experiments were conducted under controlled laboratory conditions, which do not fully replicate real-world environmental conditions. The focus was primarily on the short-term properties of the hydraulic lime mortar with metakaolin, and long-term performance was not comprehensively addressed. Future research should focus on long-term durability studies to evaluate the performance of hydraulic lime mortar with metakaolin under various environmental conditions, including weathering, freeze–thaw cycles, and chemical attacks. Field trials on actual historic structures would provide insights into the practical applicability of these materials. Further optimization of the mix ratios and additives could enhance specific properties, such as elasticity, thermal resistance, and acoustic insulation. It is also important to assess the environmental impact and sustainability of using metakaolin in lime mortars through lifecycle assessment. Comparative studies with other restoration materials can establish the relative advantages and best practices for historic building restoration.

## Figures and Tables

**Table 1:** Indexes of cement.

Whiteness %	Fineness %	Standard Consistency %	Setting Time (min)		Flexural Strength (MPa)		Compressive Strength (MPa)	
			Initial Setting	Final Setting	3 d	28 d	3 d	28 d
90.5	1.8	28.51	184	234	5.1	7.4	26.6	41.7

**Table 2:** Main chemical composition of white cement.

Oxide	CaO	SiO_2_	MgO	Al_2_O_3_	K_2_O	Fe_2_O_3_
Mass Fraction (%)	67.96	17.21	9.34	1.43	0.82	0.28

**Table 3:** Main chemical composition of MK.

Oxide	SiO_2_	Al_2_O_3_	TiO_2_	Fe_2_O_3_	CaO	K_2_O
Mass Fraction (%)	54.42	42.68	1.77	0.54	0.23	0.13

**Table 4:** Main chemical composition of hydrated lime.

Oxide	CaO	MgO	SiO_2_	Al_2_O_3_	K_2_O	Fe_2_O_3_
Mass Fraction (%)	97.11	1.20	0.86	0.35	0.19	0.12

**Table 5:** The mixing proportions of the test.

Test No.	White Cement (%)	Lime (%)	Heavy Calcium Carbonate (%)	MK (%)	Cement–Sand Ratio	Water–Cement Ratio
LPSMK0	35.0	40.0	25.0	0	1:2	0.60
LPSMK1	35.0	37.0	25.0	3.0		
LPSMK2	35.0	34.0	25.0	6.0		
LPSMK3	35.0	31.0	25.0	9.0		
LPSMK4	35.0	28.0	25.0	12.0		

**Table 6:** Elastic modulus and Poisson’s ratio of HLM.

Test No.	MK (%)	Elastic Modulus (GPa)	Poisson’s Ratio
LPSMK0	0	2.19	0.253
LPSMK1	3.0	4.35	0.194
LPSMK2	6.0	4.57	0.156
LPSMK3	9.0	6.09	0.123
LPSMK4	12.0	7.88	0.107

## Data Availability

The original contributions presented in the study are included in the article, further inquiries can be directed to the corresponding author.

## References

[1] Ornelas C., Sousa F., Guedes J.M., Breda-Vázquez I. (2023). Monitoring and Assessment Heritage Tool: Quantify and classify urban heritage buildings. Cities.

[2] Munarim U., Ghisi E. (2016). Environmental feasibility of heritage buildings rehabilitation. Renew. Sustain. Energy Rev..

[3] Shan M., Chen Y.-F., Zhai Z., Du J. (2022). Investigating the critical issues in the conservation of heritage building: The case of China. J. Build. Eng..

[4] Guzmán P.C., Roders A.R.P., Colenbrander B.J.F. (2017). Measuring links between cultural heritage management and sustainable urban development: An overview of global monitoring tools. Cities.

[5] Aggelakopoulou E., Bakolas A., Moropoulou A. (2011). Properties of lime–metakolin mortars for the restoration of historic masonries. Appl. Clay Sci..

[6] Kang S.-H., Lee S.-O., Hong S.-G., Kwon Y.-H. (2019). Historical and Scientific Investigations into the Use of Hydraulic Lime in Korea and Preventive Conservation of Historic Masonry Structures. Sustainability.

[7] Elert K., Rodriguez-Navarro C., Sebastián E., Hansen E., Cazalla O. (2002). Lime Mortars for the Conservation of Historic Buildings Author(s). Stud. Conserv..

[8] Vavričuk A., Bokan-Bosiljkov V., Kramar S. (2018). The influence of metakaolin on the properties of natural hydraulic lime-based grouts for historic masonry repair. Constr. Build. Mater..

[9] Silva B.A., Pinto A.P.F., Gomes A. (2014). Influence of natural hydraulic lime content on the properties of aerial lime-based mortars. Constr. Build. Mater..

[10] Grilo J., Silva A.S., Faria P., Gameiro A., Veiga R., Velosa A. (2014). Mechanical and mineralogical properties of natural hydraulic lime-metakaolin mortars in different curing conditions. Constr. Build. Mater..

[11] Garijo Alonso L., Zhang X., Ruiz G., Ortega J.J. (2019). Age effect on the mechanical properties of natural hydraulic and aerial lime mortars. Constr. Build. Mater..

[12] Shaoyun Z., Manli S., Qinglin G., Linyi Z., Zhipeng L. (2023). Study on the Mechanical Properties and Durability of Hydraulic Lime Mortars Based on Limestone and Potassium Feldspar. Appl. Sci..

[13] Luo K., Li J., Lu Z., Jiang J., Niu Y. (2019). Effect of nano-SiO_2_ on early hydration of natural hydraulic lime. Constr. Build. Mater..

[14] Moon K.Y., Cho J.S., Choi M.K., Cho K.H., Ahn J.W., Hong C.W., Ur S.C. (2016). Effect of blast furnace slag on the hydration properties in natural hydraulic lime. J. Ceram. Process. Res..

[15] Bumanis G., Vitola L., Stipniece L., Locs J., Korjakins A., Bajare D. (2020). Evaluation of Industrial by-products as pozzolans: A road map for use in concrete production. Case Stud. Constr. Mater..

[16] Fapohunda C., Akinbile B., Shittu A. (2017). Structure and properties of mortar and concrete with rice husk ash as partial replacement of ordinary Portland cement—A review. Int. J. Sustain. Built Environ..

[17] Tafraoui A., Escadeillas G., Vidal T. (2016). Durability of the Ultra High Performances Concrete containing metakaolin. Constr. Build. Mater..

[18] Andrejkovicová S., Velosa A.L., Ferraz E., Rocha F. (2014). Influence of clay minerals addition on mechanical properties of air lime-metakaolin mortars. Constr. Build. Mater..

[19] Navrátilová E., Rovnaníková P. (2016). Pozzolanic properties of brick powders and their effect on the properties of modified lime mortars. Constr. Build. Mater..

[20] Siddiqua A., Hahladakis J.N., Al-Attiya W. (2022). An overview of the environmental pollution and health effects associated with waste landfilling and open dumping. Env. Sci. Pollut. Res. Int..

[21] Albidah A., Alghannam M., Abbas H., Almusallam T., Al-Salloum Y. (2021). Characteristics of metakaolin-based geopolymer concrete for different mix design parameters. J. Mater. Res. Technol..

[22] Zhang D.J., Zhao J.H., Wang D.M., Wang Y.R., Ma X.D. (2020). Influence of pozzolanic materials on the properties of natural hydraulic lime based mortars. Constr. Build. Mater..

[23] Brooks J.J., Megat Johari M.A. (2001). Effect of metakaolin on creep and shrinkage of concrete. Cem. Concr. Compos..

[24] Al-Hashem M., Amin M., Ajwad A., Afzal M., Khan K., Faraz M., Qadir M., Khan H. (2022). Mechanical and Durability Evaluation of Metakaolin as Cement Replacement Material in Concrete. Materials.

[25] Silva B.A., Pinto A.P.F., Gomes A. (2015). Natural hydraulic lime versus cement for blended lime mortars for restoration works. J. Constr. Build. Mater..

[26] (2005). Test Method for Fluidity of Cement Mortar.

[27] Alvarez J., Veiga M., Martínez-Ramírez S., Secco M., Faria P., Maravelaki-Kalaitzaki P., Ramesh M., Papayianni I., Valek J. (2021). RILEM TC 277-LHS report: A review on the mechanisms of setting and hardening of lime-based binding systems. Mater. Struct..

[28] Mohammadifar L., Miraki H., Rahmani A., Jahandari S.S., Mehdizadeh Miyandehi B., Rasekh H., Samadi P., Samali B. (2022). Properties of Lime-Cement Concrete Containing Various Amounts of Waste Tire Powder under Different Ground Moisture Conditions. Polymers.

[29] Mehdipour I., Razzaghi M.S., Amini K., Shekarchi M. (2013). Effect of mineral admixtures on fluidity and stability of self-consolidating mortar subjected to prolonged mixing time. Constr. Build. Mater..

[30] (2011). Test Methods for Water Requirement of Normal Consistency, Setting Time Andsoundness of the Portland cement.

[31] Andrejkovičová S., Maljaee H., Rocha D., Rocha F., Soares M., Velosa A. (2022). Mortars for Conservation of Late 19th and Early 20th Century Buildings—Combination of Natural Cements with Air Lime. Materials.

[32] El-Diadamony H., Amer A.A., Sokkary T.M., El-Hoseny S. (2019). Hydration and characteristics of metakaolin pozzolanic cement pastes. HBRC J..

[33] Zhao Y., Zhang Y. (2023). A Review on Hydration Process and Setting Time of Limestone Calcined Clay Cement (LC3). Solids.

[34] (2009). Standard for Test Methods of Basic Properties of Building Mortar.

[35] Chi M., Huang R. (2012). Effect of montmorillonite as additive on the properties of cement-based composites. Sci. Eng. Compos. Mater..

[36] Ince C., Carter M.A., Wilson M.A. (2015). The water retaining characteristics of lime mortar. Mater. Struct..

[37] Mahfouz T., Khedr S., Abdel-Mooty M. (2009). Evaluation of lime mortars for the repair of historic buildings. Structural Studies, Repairs and Maintenance of Heritage Architecture XI.

[38] Pan Z., Sanjayan J.G., Rangan B.V. (2009). An investigation of the mechanisms for strength gain or loss of geopolymer mortar after exposure to elevated temperature. J. Mater. Sci..

[39] Hassannezhad K., Akyol Y., Dursun M.C., Ow-Yang C.W., Gulgun M.A. (2022). Effect of Metakaolin and Lime on Strength Development of Blended Cement Paste. Constr. Mater..

[40] Kozlovcev P., Valek J. (2021). The micro-structural character of limestone and its influence on the formation of phases in calcined products: Natural hydraulic limes and cements. Mater. Struct..

[41] Frías M., Cabrera J. (2001). Influence of MK on the Reaction Kinetics in MK/lime and MK-Blended Cement Systems at 20 °C. Cem. Concr. Res..

[42] Schuab M., Santos W., Ribeiro Borges P. (2021). On the development of MK/BFS alkali-activated materials as repair mortars: Performance under free and restrained shrinkage tests. Constr. Build. Mater..

[43] (2015). Test Methods for Water Vapour Transmission Properties of Building Materials and Products.

[44] (2021). Test Method for Strength of Cement Mortar (ISO Method).

[45] Du H., Pang S. (2020). High-performance concrete incorporating calcined kaolin clay and limestone as cement substitute. Constr. Build. Mater..

[46] Afzal Q., Abbas S., Abbass W., Ahmed A., Azam R., Riaz M.R. (2020). Characterization of sustainable interlocking burnt clay brick wall panels: An alternative to conventional bricks. Constr. Build. Mater..

